# Predicting the spread and persistence of genetically modified dominant sterile male mosquitoes

**DOI:** 10.1186/s13071-021-04982-1

**Published:** 2021-09-16

**Authors:** Adrien Ickowicz, Scott D. Foster, Geoffrey R. Hosack, Keith R. Hayes

**Affiliations:** CSIRO Data61, Hobart, Australia

**Keywords:** Entomological survey data, Sterile male, Expert elicitation, Bayesian hierarchical model, Monitoring

## Abstract

**Background:**

Reproductive containment provides an opportunity to implement a staged-release strategy for genetic control of malaria vectors, in particular allowing predictions about the spread and persistence of (self-limiting) sterile and male-biased strains to be compared to outcomes before moving to (self-sustaining) gene-drive strains.

**Methods:**

In this study, we: (i) describe a diffusion–advection–reaction model of the spread and persistence of a single cohort of male mosquitoes; (ii) elicit informative prior distributions for model parameters, for wild-type (WT) and genetically modified dominant sterile strains (DSM); (iii) estimate posterior distributions for WT strains using data from published mark-recapture-release (MRR) experiments, with inference performed through the Delayed-Rejection Adaptive Metropolis algorithm; and (iv) weight prior distributions, in order to make predictions about genetically modified strains using Bayes factors calculated for the WT strains.

**Results:**

If a single cohort of 5000 genetically modified dominant sterile male mosquitoes are released at the same location as previous MRR experiments with their WT counterparts, there is a 90% probability that the expected number of released mosquitoes will fall to < 1 in 10 days, and that by 12 days there will be a 99% probability that no mosquitoes will be found more than 150 m from the release location.

**Conclusions:**

Spread and persistence models should form a key component of risk assessments of novel genetic control strategies for malaria vectors. Our predictions, used in an independent risk assessment, suggest that genetically modified sterile male mosquitoes will remain within the locality of the release site, and that they will persist for a very limited amount of time. Data gathered following the release of these mosquitoes will enable us to test the accuracy of these predictions and also provide a means to update parameter distributions for genetic strains in a coherent (Bayesian) framework. We anticipate this will provide additional insights about how to conduct probabilistic risk assessments of stage-released genetically modified mosquitoes.

**Graphical abstract:**

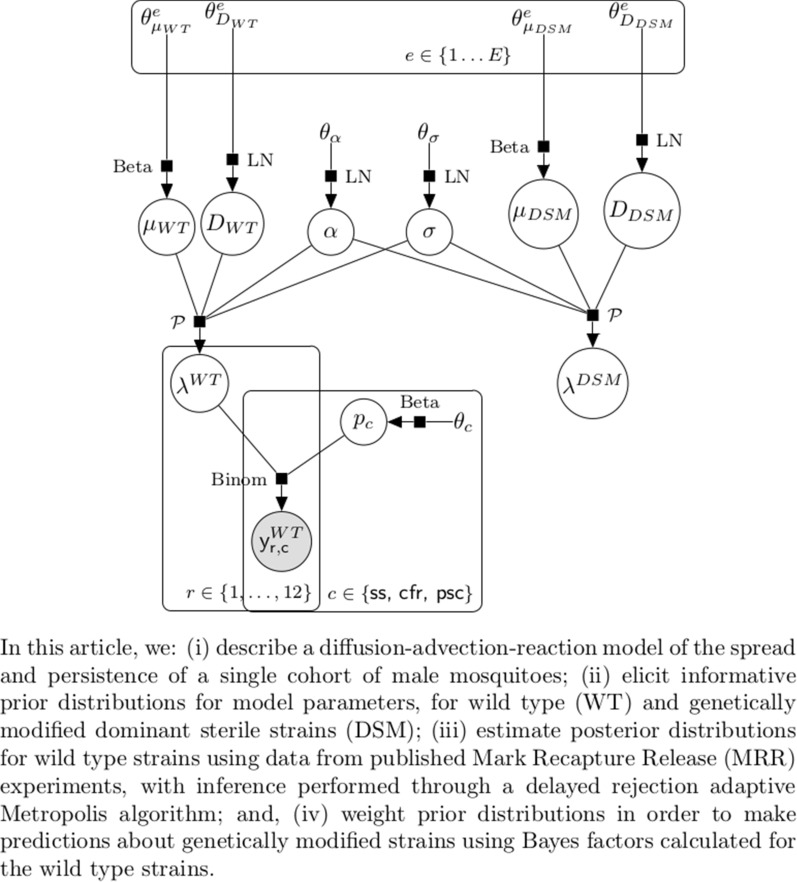

**Supplementary Information:**

The online version contains supplementary material available at 10.1186/s13071-021-04982-1.

## Background

Over the last 30 years conventional control strategies have reduced the global incidence rate of malaria. The efficacy of these strategies, however, appears to be waning. Global malaria incidence rates have barely changed since 2014, and progress has stalled at around 57 cases per 1000 population at risk despite increased expenditure on elimination and prevention between from 2014 to 2017 [1]. Consequently, the World Health Organisation predicts that morbidity targets for 2020, 2025 and 2030 will not to be met without significant improvements in vector control and chemoprevention.

Laboratory studies and modelling indicate that novel control strategies that use gene drives to force engineered alleles through vector populations with the aim to suppress these populations or render them unable to transmit plasmodium parasites could augment conventional strategies and significantly improve current rates of malaria control [2–5]. As with many novel technologies, however, the safety of genetic control methods is uncertain, and the potential for spread and ecosystem-wide impacts makes the gene drive strategy contentious [6, 7].

To date, all research on gene-drive modified mosquitoes (GDMMs) has been conducted in laboratories under strict physical, ecological, reproductive and/or molecular containment [8]. Current guidance recommends that if containment is to be relaxed it should be done in a “phased-release” strategy wherein data are gathered, risks are evaluated and containment is gradually lifted in a step-wise fashion if and when the risks are deemed acceptable [9, 10]. The possibility of long-distance dispersal [11] and biosecurity concerns with large semi-field enclosure, however, may erode confidence in strategies that try to gradually lift ecological or physical containment.

Genetic and reproductive containment strategies may be more amenable to a gradual-release strategy, and several approaches are currently being pursued [12–14]. In this paper we focus on the spatio-temporal dynamics that govern the spread and persistence of a single cohort of genetically modified, dominant sterile male (DSM) *Anopheles coluzzii* mosquitoes released in a small village in Burkina Faso. These sterile male mosquitoes are reproductively contained (but females that carry the sterilising construct are fertile) and could represent the first stage in a three-stage pathway to malaria vector control using a gene drive that results in a male-biased sex ratio [15, 16].

In our analysis the spatio-temporal dynamics of DSM mosquitoes are driven by: (i) a diffusion process that accounts for the dispersal capacity of the insects; (ii) an advection process that accounts for the attraction of individuals to swarms, and (iii) a reaction process that accounts for the death of sterile male mosquitoes. These processes can be described mathematically by a partial differential equation (PDE) of the advection–diffusion–reaction type, which models how the distribution of mosquitoes in time and space is affected by a chemo-attractant present in the environment. Models of this type have also been widely used within the environmental and ecological literature. In particular, such models have been used previously to model mosquitoes, for example the dispersal of *Aedes albopictus* in Reunion Island using a similar type of parabolic PDE [17].

Our objective is to predict the spread and persistence of the genetically modified mosquitoes ahead of a proposed field release. These predictions are an important component of an independent risk assessment conducted prior to the field release [18] that assessed the risk of vector-borne disease transmission [19] and could also help to inform the design of a post-release monitoring strategy. Our approach implements the Bayesian paradigm; we elicit the prior distribution of model parameters from relevant experts, for both wild-type (WT) mosquitoes and their DSM counterparts. We obtain the posterior model parameter values for the WT parameters using the results of mark-release-recapture (MRR) experiments conducted by [20]. We then use Bayesian model averaging to predict the spread and persistence of DSM mosquitoes.

## Methods

### PDE model

The literature review conducted in [21] suggests that the behaviour of host-seeking mosquitoes can be categorised as: (i) *plume finding*, in which flight direction is either random (kinesis), determined by visual features in the absence of wind or deliberately upwind, downwind or crosswind (anemotaxis) when wind is present; and, (ii) *plume tracking*, where once the mosquito detects an odour plume, flight direction is deliberately upwind, or possibly determined by the odour gradient in windless conditions, in order to find the odour source (positive chemotaxis). More details on the properties of these search strategies can be found in [22].

In this analysis, we assume that village compounds (groups of closely spaced houses) provide a source of carbon dioxide (CO_2_) that acts as an attractant for female *Anopheles gambiae* sensu strictu and *Anopheles coluzzi* mosquitoes [23, 24]. We further assume that male mosquitoes swarm in places where the probability of encountering receptive females is highest [25]; hence, swarms occur in and around compounds (as evident in Figure 1 of [20]). Consequently, high concentrations of attractant result in flights that (on average) are closer to the direction of the nearest compound for both males and females. Conversely, low concentrations have less influence on the mosquitoes and result in greater variability in flight direction. We do not incorporate the effect of wind speed or direction into the analysis (rather, we assume that the local dispersal dynamics are not greatly affected by the wind at this spatial scale), and we also assume that the attraction process is not influenced by the number of mosquitoes.

With these assumptions we developed an advection–diffusion–reaction model to describe the expected number of male WT and DSM mosquitoes,

1 $$\lambda _{\varvec{t}}= {D}\left [ \lambda _{\varvec{s}\varvec{s}}-{\alpha }\cdot \nabla _{\varvec{s}} \left( \lambda U(\varvec{s})\right) \right ]-{\mu }\lambda,$$

with the following boundary conditions (Neumann reflecting conditions):

2 $$\frac{\partial \lambda }{\partial s}= 0$$

where $U(\varvec{s})$ is a function that describes the strength of mosquito attraction at location *s*, and ${\alpha }$ enables us to modulate the effect of the utility function on the overall attraction. Utility functions like this have been used in similar ecological contexts to describe an attractive or repulsive flux [26].

In this model we use swarm sites as centers of attraction and assume that the strength of attraction decays according to a squared-sum exponential decay kernel

3 $$U(\varvec{s})= \sum _{\varvec{s}_\ell \in \mathcal {L}} \exp \left [ - \frac{|\varvec{s}- \varvec{s}_\ell |^2}{\sigma ^2}\right ]$$

where $\mathcal {L}$ are the known swarm locations (identified prior to the MRR experiment [20]) and $\sigma$ is an unknown parameter that controls the range of attraction—that is, the distance beyond which the attraction of a swarm site $\varvec{s}_\ell$ for a mosquito is negligible.

The theoretical solution to the partial differential Eq. 1 is the expected number of male mosquitoes at time *t* and location $\varvec{s}=(\text {easting},\text {northing})$. The partial derivatives are denoted $\lambda _{\varvec{t}}$ (with respect to time) and $\lambda _{\varvec{s}\varvec{s}}$ (with respect to location). Because we use a numerical solver, we define a grid over the spatial domain. Each grid cell is therefore one areal unit. In this study, an areal unit is a square of 100 m^2^ of area. All process model parameters are summarised in Table 1.

**Table 1:** The parameters and units of the diffusion–advection–reaction model for sterile male mosquitoes

Name	Description	Units
${\alpha }$	Attractiveness of swarms	reward^−1^ $\text{m}^{-1}$
${\mu }$	Male mosquito mortality rate	day$^{-1}$
$\lambda$	The expected number of male mosquitoes	Number of mosquitoes per areal unit
${D}$	Isotropic diffusion coefficient	$\text{m}^2$ day$^-1$
$\sigma$	Decay of attraction to swarm sites	m

### Data

In this analysis we use the results of five MRR experiments [20] wherein approximately 5000 adult male WT mosquitoes, marked with a coloured powder, were released 2 h before swarming (around 16:00 h), at three different locations, on five different dates (Table 2). Mosquitoes were recaptured in three ways via: (i) swarm sampling in the evening (times were not specified, but we assume the sampling occurred between 19:00 and 21:00 h); (ii) pyrethroid spray catches (PSC) in the morning inside houses; and (iii) placement of humidified clay pots within rooms within houses, which were subsequently checked in the morning (between 06:00 and 07:00 h). All release and recapture sites were geo-located to allow for a spatial model to be used.

**Table 2:** Summary of the MRR experiments conducted by Epopa et al. [20] in Burkina Faso

MRR	Date	Release site	GPS coordinates		*N* mosquitoes released	*N* mosquitoes captured	Distance (m)
			Longitude	Latitude			
1	2013-10-09	A	− 4.4724	11.2347	1146	32	140
1		B	− 4.4755	11.2342	1158	9	344
1		C	− 4.4718	11.2318	1103	6	205
2	2014-05-07	A	− 4.4724	11.2347	1878	6	94
2		B	− 4.4755	11.2342	1655	1	266
2		C	− 4.4718	11.2318	1734	1	193
3	2014-09-04	A	− 4.4724	11.2347	1665	56	133
3		B	− 4.4755	11.2342	1673	4	440
3		C	− 4.4718	11.2318	1684	13	205
4	2015-04-09	A	− 4.4724	11.2347	2107	1	55
4		B	− 4.4755	11.2342	2013	3	386
4		C	− 4.4718	11.2318	1953	18	190
5	2015-10-09	C	− 4.4724	11.2347	5992	18	141

The last column presents the average distance for recapture (in meters). We use the results of the first four experiments to parameterise our model and calculate Bayes factor for expert-derived priors, and use the fifth MRR experiment to validate the model predictions

Recaptures were performed for 7 days after release for all experiments except the first one where recaptures were performed for 5 days. We used the results of the first four MRR experiments to derive posterior estimates of male WT model parameters, reserving the results of the fifth experiment to compare with model predictions.

### Priors

#### Process model

A key challenge when conducting risk assessments of a novel technology is the lack of empirical information. Without any operational history from which success and failure rates might be estimated, quantitative risk predictions must (at least initially) be based on the testable predictions of domain experts. The Bayesian inference paradigm encourages the careful elicitation of prior information and provides a coherent mechanism for updating this information as data become available [27]. The experimental observations and expert elicitation, however, must be independent but carefully aligned for this process to work.

For this analysis we carefully elicited opinions on the mortality rate and dispersal distance of WT and DSM male mosquitoes from four independent recognised experts on malaria transmission by mosquitoes in Africa and genetic vector control methods; the independent experts were not involved in the development of the DSM mosquitoes [18]. The experts were encouraged to draw on their own research experience and knowledge of the published literature while constructing their assessments. Prior to the elicitation, experts were provided information on the genetic construct (originally incorporated into the G3 laboratory colony strain of *Anopheles gambiae*) and were told that the genetically modified strain would be repeatedly backcrossed with WT mosquitoes captured in the vicinity of a biosecure laboratory in order to gradually replace the G3 genetic background with that of the locally originated strain. The location of the laboratory was provided to each expert, but the species of local WT mosquitoes was not specified. For the mortality rate, we proposed that the experts answer questions about either the probability of mortality, the probability of survival or the life expectancy. All responses were converted to an estimate of the daily probability of survival (*p*) which was then converted to the mortality rate parameter ($\mu = -\log (p)$) used in the PDE model (Additional file 1).

The model’s diffusion parameter (${D}$) was not elicited directly but calculated using the experts’ prior opinion on the average daily dispersal distance (*d*) of male mosquitoes. All experts agreed that a log-normal distribution provided the most appropriate description of their uncertainty for both WT and DSM strains. The model’s prior diffusion parameter was subsequently calculated using the relation ${D}= d^2/\pi t$ (see Additional file 2 for a detailed derivation). The geographical and temporal context of the elicitation was carefully prescribed, including a standardized time frame (dispersal over a 1-day period), so that all experts’ opinions could be combined.

The model’s two chemotaxis-related parameters are: (i) the relative strength of attraction towards the source of the attractant that a mosquito experiences at a location (${\alpha }$), with units $\text{m}^{-1} \text{R}^{-1}$ where m is the distance in meters between the location and the source and R is a measure of the “reward” at the source, often expressed as the concentration of CO_2_ for female mosquitoes; and (ii) the distance beyond which a mosquito is negligibly attracted to a swarm site, called the range ($\sigma$) with units m. The attractiveness of a swarm site at distance $\sigma$ from a mosquito is roughly 38% of its attractiveness at zero distance.

Finding prior information on these parameters proved difficult for the particular setting of this model, in particular for ${\alpha }$. We therefore chose a very broad prior distribution for ${\alpha }$ that spans several orders of magnitude, from $1\%$ (1st percentile) to $140\%$ (99th percentile) of the attraction at the source. For the range $\sigma$ defined the same way we do, different articles on related species provide values ranging from 3 [28] to 18 m for single baiting or > 36 m for double baiting [29], or even 40 m [30]. We therefore used a log-normal distribution for this prior with positive probability across all these possibilities (Table 3).

**Table 3:** Process (PDE model) and observation model parameters, prior distributions and sources

Parameter	Prior parameters	Prior distribution	Source
Mortality ($\mu$)	$\theta _\mu$: mixture (see Fig. 2)	Beta	Experts
Diffusion (*D*)	$\theta _D$: mixture (see Fig. 2)	Log-normal	Experts
Swarm attraction ($\alpha$)	$\theta _\alpha = \{ -2, 1 \}$	Log-normal	Weakly informative
Swarm range ($\sigma$)	$\theta _\sigma = \{3.5, 4 \}$	Log-normal	Literature
Catchability cfr ($p_{\text{cfr}}$)	$\theta _{\text{cfr}} =\{1, 1\}$	Beta	Weakly informative
Catchability psc ($p_{\text{psc}}$)	$\theta _{\text{psc}} =\{1.4, 1\}$	Beta	SOP
Catchability SS ($p_{\text{ss}}$)	$\theta _{\text{ss}} =\{10, 20\}$	Beta	SOP

*cfr* Clay pots resting catches,* psc* pyrethroid spray catches,* SS* swarming samples,* PDE* partial differential equation,* SOP* standard operating procedures (the procedure used during the MRR experiments are described in a set of SOP that were made available to us as part of an independent risk assessment process)

#### Observation model

An important advantage of the Bayesian paradigm is that it allows us to develop more realistic hierarchical models that capture uncertainty in the biological and physical processes that drive a phenomenon, as well as the uncertainty associated with the measurement process with which these processes are observed. In this context, this allows us to reflect the uncertainty about how many mosquitoes any particular trap will catch given a known number of mosquitoes in the proximity of the trap.

Here we represent the observation model uncertainty using three, trap-specific, catchability parameters, denoted $p_{\text{cfr}}$ for clay-pots, $p_{\text{psc}}$ for insecticide spraying and $p_{\text{ss}}$ for swarm sampling. These parameters define the probability of catching a mosquito given that it was in close proximity of the trap at the time of sampling [31]. Note that prior and posterior estimates of these parameters depend on the model resolution because the definition of “close proximity” is determined by the resolution of the raster (i.e. the grid cell size) over which the PDE model is numerically solved. Hence, all predictions must be made at the same resolution used for solving the PDE and updating the prior distributions.

The priors for the catchability parameters were chosen according to the following principles:

- For $p_{\text{ss}}$, a beta distribution with parameters $\alpha_{\text{ss}} = 10$ and $\beta_{\text{ss}} = 20$ (meaning on average one third of the mosquitoes in the swarm are caught, following the standard operating procedures of the MRR experiment, which requires one third of the swarm to be captured).
- For $p_{\text{psc}}$, a beta distribution with parameters $\alpha_{\text{psc}} = 1.4$ and $\beta_{\text{psc}} = 1$. This prior was chosen because the PSC procedure involves spraying an entire room and capturing all the dead mosquitoes in the room. The PDE model, however, is specified at a resolution of the compounds, which on average have 1.4 rooms. We assume that each room has on average the same number of male mosquitoes.
- For $p_{\text{cfr}}$, assuming this is the least efficient method but not having more information we deliberately selected a fairly low informative prior. So we chose a beta distribution with parameters $\alpha_{\text{cfr}} = 1$ and $\beta_{\text{cfr}} = 1$.

A complete list of process and observation model priors and their sources is summarised in Table 3.

### Inference

#### WT male parameters

A Bayesian hierarchical model (BHM) is classically written:

4 $${\varvec{\pi }}(\theta , \lambda | \mathsf{y})\propto \underbrace{{\varvec{\pi }}(\mathsf{y} | \lambda )}_{\text{data}} \underbrace{{\varvec{\pi }}(\lambda | \theta )}_{\text{process}} \underbrace{{\varvec{\pi }}( \theta )}_{\text{prior}}$$

where here $\theta$ is the vector of parameters of the PDE model and the trap catchability parameters, $\lambda$ refers to the expected abundance of male mosquitoes in the vicinity of the traps and *y* refers to the MRR data collected following the 12 releases ($r = {1, \dots , 12}$), observed with three different collection (trap) techniques ($c = {1, \dots , 3}$). Assuming conditional independence of the observations, the posterior distribution can be re-written as:

5 $${\varvec{\pi }}(\theta _d, {\lambda _r}, {p_c} | \mathsf{y})\propto \prod _c \prod _r \left [ \underbrace{{\varvec{\pi }}(\mathsf{y_{r,c}} | \lambda _r, p_c)}_{\text{data}} \underbrace{{\varvec{\pi }}(\lambda _r | \theta _d)}_{\text{process}} \right ] \underbrace{{\varvec{\pi }}( \theta _d, p_c)}_{\text{prior}}$$

where $\theta _d$ is the list of parameters defined in Table 1.

For a given release *r*, we describe the number of available released mosquitoes at time *t* as a Poisson random variable with expectation $\lambda _r(t)$, where the dependence of this expectation on location is suppressed:

6 $$n_r(t) | \lambda _r(t)\sim \text {Poisson}(\lambda _r(t))$$

where $\lambda _r(t)$ is given by the PDE model described in Eq. 1. The data $\mathsf{y}$ are the observed count of male mosquitoes in a given trap. Since the traps catch only a portion of the mosquitoes that occur in their immediate vicinity, a natural trap observation model is the binomial distribution:

7 $$\begin{aligned} {\mathsf{y}_\mathsf{r,c}} | n_r, p_c\sim & {} \text {Binomial}(n_r, p_c) \end{aligned}$$

where $n_r$ is a realization of the Poisson random variable $\lambda _r$ (the expected number of released mosquitoes in the trap’s immediate vicinity), and $p_c$ is the catchability parameter, or “trap efficiency”, which is trap dependent. In this study we do not account for the possibility of false positive (insects or mosquito species incorrectly identified as released male mosquitoes) or false negative (released male *An. gambiae* mosquitoes incorrectly identified as something else) probabilities as they are assumed to be equal to zero [20].

Given the binomial likelihood, the posterior distribution is obtained by integrating over the possible Poisson outcomes:

8 $${\varvec{\pi }}(\theta _d, \lambda _r, {p_c} | \mathsf{y})\propto \prod _c \prod _r \left [ \sum _{n_r} \underbrace{{\varvec{\pi }}(\mathsf{y_{r,c}} | n_r, p_c) {\varvec{\pi }}(n_r | \lambda _r)}_{\text{data}} \right ] \underbrace{{\varvec{\pi }}(\lambda _r | \theta _d)}_{\text{process}} \underbrace{{\varvec{\pi }}( \theta _d)}_{\text{prior}}$$

Markov Chain Monte Carlo (MCMC) inference methods have been successfully applied to hierarchical Bayesian models, similar to the model described here, on many occasions (see, for example [32–35]). We found, however, that the standard random-walk Metropolis MCMC routine was too slow to mix. Furthermore, in this context, more advanced methods, such as Hamiltonian Monte Carlo sampling, which require repeated likelihood computations along the proposal path, are inefficient because of the high computational costs entailed by the need to numerically solve the PDE model for each new proposal.

We found a reasonable compromise through the Delayed-Rejection Adaptive Metropolis (DRAM) algorithm, proposed by [36]. This MCMC algorithm uses a multivariate proposal distribution that automatically adapts to allow for posterior correlations between the parameters and identifies the directions of principal change along the ridges in the posterior surface. The acceptance rate of the DRAM algorithm is also improved by using a delayed rejection scheme where, instead of immediately advancing the chain following rejection of a parameter set, a second proposal is made that depends on both the current position of the chain and the rejected parameter set. We implemented DRAM by using the function modMCMC in the FME package [37] in R ([38]; https://www.R-project.org/), using one delayed rejection step and updating the proposal distribution every 200 iterations. We run a total of three MCMC chains of 15000 samples each, with 5000 used as burn-in. The convergence of the chains was assessed using Gelman’s $\hat{R}$ criterion (see chapter 11 in [39]).

### Posterior prediction validation

We evaluated the accuracy of the posterior model predictions by comparing them against the observed recaptures in the MRR experiment that we deliberately withheld from the inference procedure. The fifth MRR experiment was conducted at the same location as the first four experiments, and under similar conditions with two exceptions: (i) all marked male mosquitoes were released at a single location, and (ii) a much larger number of mosquitoes were released (Table 2). The experimental conditions and population dynamics might therefore be considered to be slightly different than those which prevailed during the first four experiments. If the model is nonetheless able to make sufficiently accurate predictions, then we may be more confident in its ability to be generalised to other similar release scenarios.

### Bayesian model averaging

The field data for the WT male mosquito allows us to calculate the posterior distribution for the WT mortality and dispersal distance parameters. This in turn allows us to measure how well each expert’s prior distribution matches the posterior distribution, and weight each expert’s response accordingly. We do this by considering each expert’s prior as an alternative model and use the theory of model evidence [40] to calculate the Bayes factor [19, 41].

**Fig. 1:**
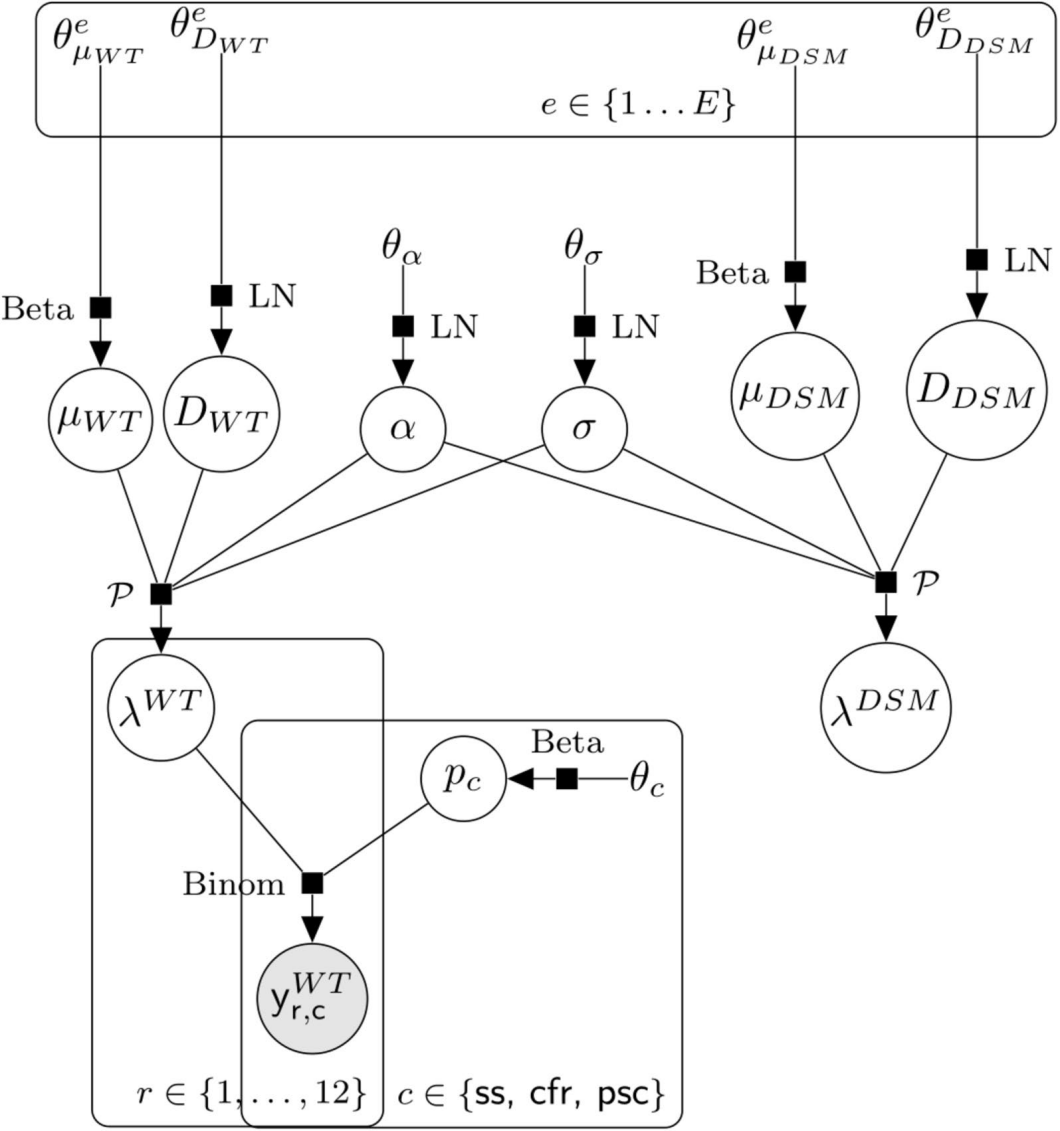
Statistical model structure. Hierarchical structure of the model for the observations on WT males and the connection to the spread and persistence prediction for DSM mosquitoes. The parameters $\alpha$ (chemotaxis attraction strength) and $\sigma$ (chemotaxis attraction range) are shared between the two strains. Abbreviations: DSM, Dominant sterile male; WT, wild type

We assume that experts who are good at predicting outcomes with WT mosquitoes (i.e. those whose prior distributions are close to the posterior distributions) will also be good at predicting outcomes with DSM mosquitoes, and weight the experts’ DSM priors by the posterior probability of the models using Bayesian model averaging to obtain a weighted linear pool of expert opinion for the DSM mortality and dispersal distance (Fig. 1).

## Results

### WT parameters

**Fig. 2:**
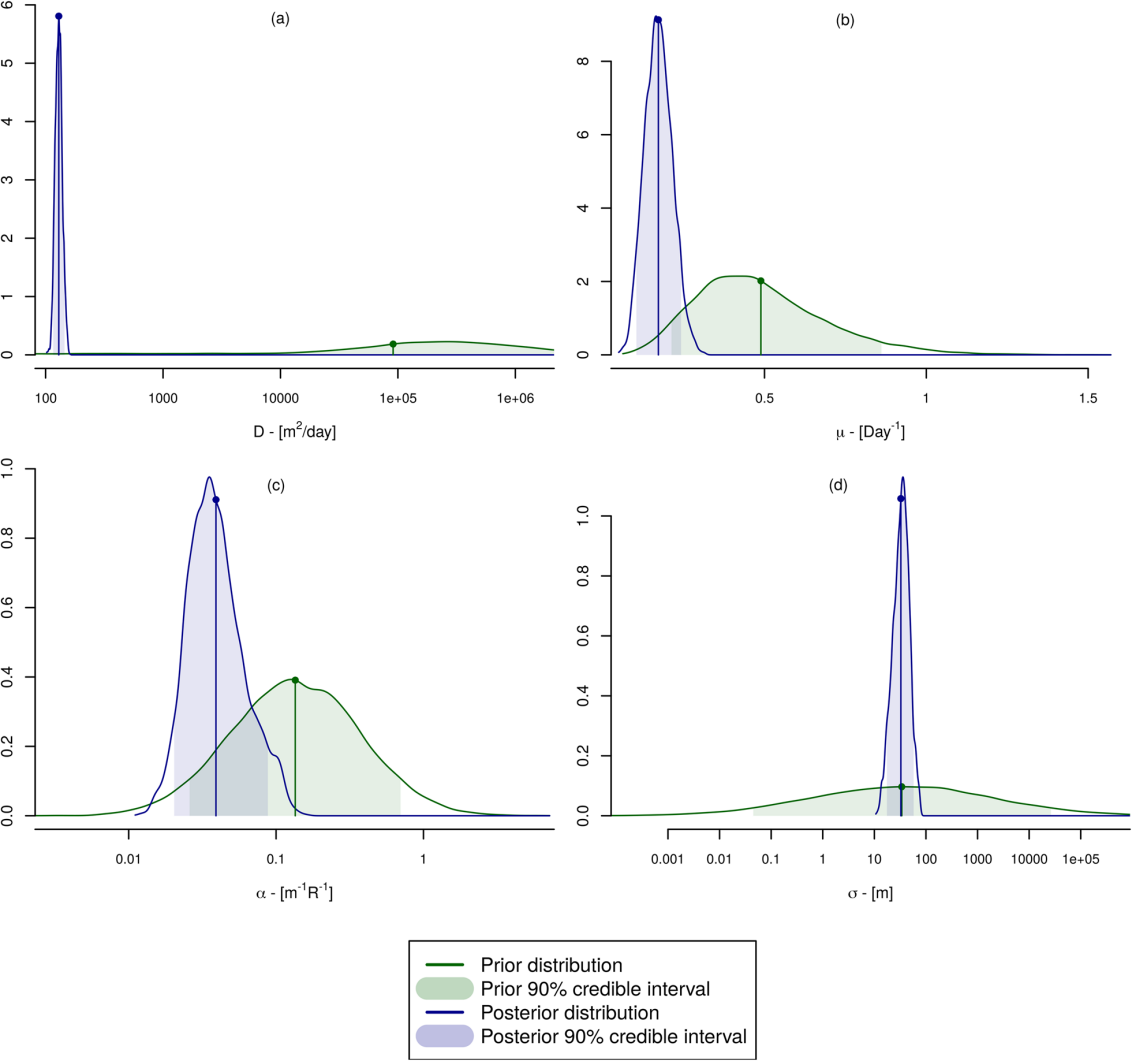
Prior and posterior distributions. Plot of the prior (elicited) and posterior (inferred) distributions for the four partial differential equation model parameters ($\alpha$, $\mu$, $\sigma$, *D*). **a** Dispersal parameter *D*, **b** daily mortality $\mu$, **c** swarm attractiveness $\alpha$, **d** swarm attractiveness decay $\sigma$

Table 4 provides the summary statistics of the posterior distributions of the PDE model parameters (Fig. 2 provides both the priors and posteriors for comparison). The use of the MRR data allowed us to provide refined estimates for the parameters of the PDE model. The diffusion coefficient in particular has seen its uncertainty decrease, yielding a mean value of 127 m^2^ per day. The chemotaxis component has a posterior mean value of about 0.07, while the range parameter for the mosquito attraction is about 33.9 m.

**Table 4:** Summary statistics for the posterior distributions of the PDE model parameters inferred from the wild-type MRRs

Parameter	Mean	Q05	Q95
Mortality ($\mu$)^a^	0.16 (0.14)	0.11 (0.10)	0.24 (0.21)
Diffusion (*D*)	127.0	113.7	140.7
Swarm attraction ($\alpha$)	0.07	0.03	0.10
Swarm range ($\sigma$)	33.9	19.8	56.1
Catchability PSC ($p_{\text{psc}}$)	0.18	0.11	0.25
Catchability cfr ($p_{\text{cfr}}$)	0.03	0.02	0.05
Catchability SS ($p_{\text{ss}}$)	0.29	0.24	0.34

^a^The equivalent daily mortality rate value is given in parentheses, for ease of comparison with other studies. Q05: 5th quantile, Q95: 95th quantile

The posterior samples of the dispersal and swarm attraction parameters are highly correlated such that high dispersal values are associated with low attraction, and vice-versa. We anticipated this and deliberately chose a weakly informative prior for the attraction parameter to allow the data to drive their posterior estimates as far as possible. The largest observable dispersal, however, is clearly dictated by the distance between the sources of attractant (compounds) and the traps laid out in the field. In this instance, all traps were laid within 500 m of release sites and compounds (Fig. 1 in [20]), limiting the ability to infer the possibility of the much higher dispersal values represented in the expert prior (Fig. 2).

### Spread and persistence of male WT mosquitoes

**Fig. 3:**
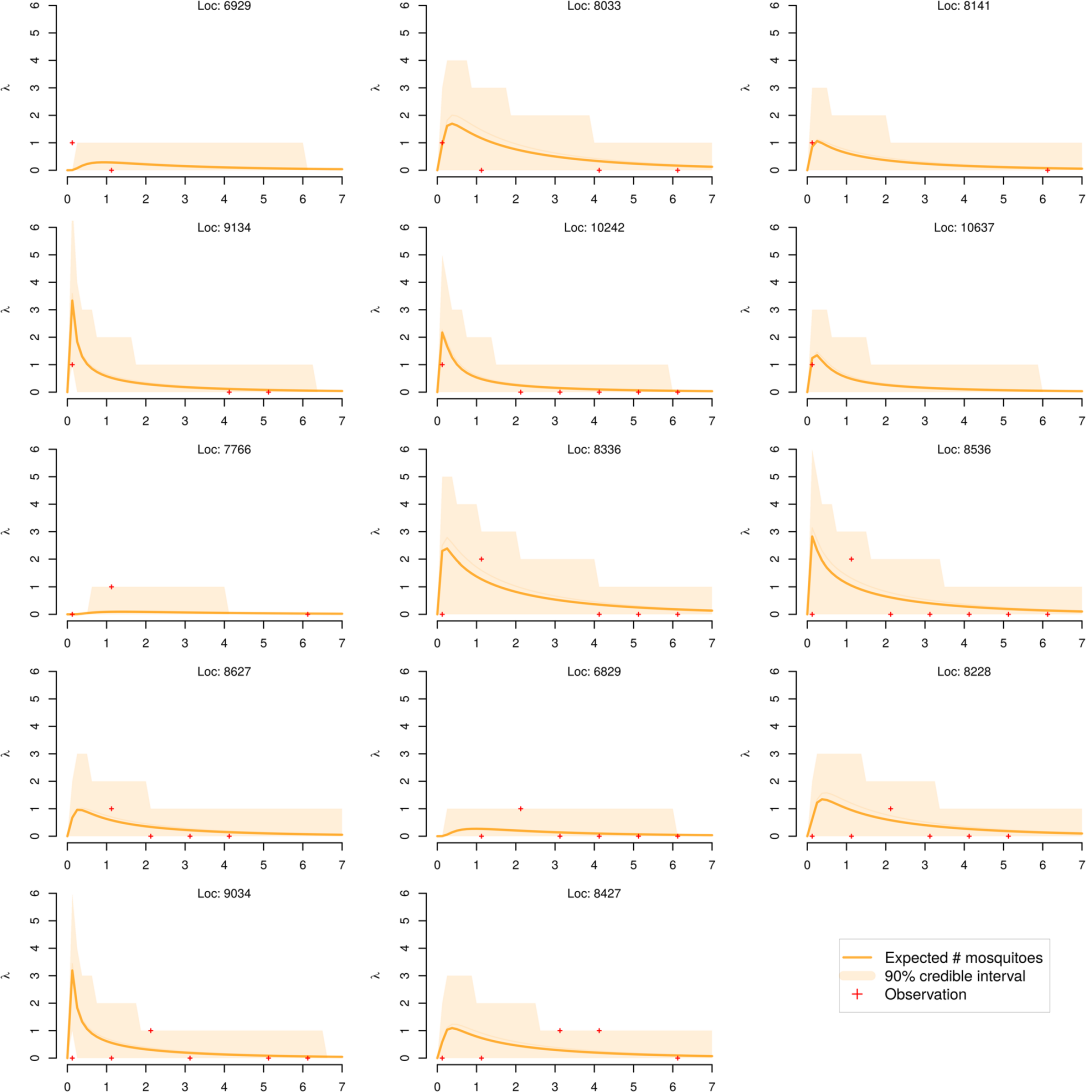
Model performance. Plot of the true observations (red crosses) and the posterior predictive expected number of catches (orange line) from the simulated model, as a function of days. The orange shading represents the 90% credible interval for the number of catches at the specified location. It is expected that 90% of the red crosses fall within the orange polygon

Figure 3 provides an overview of the expected evolution of the number of mosquitoes caught in the set of traps set for the fifth MRR (just 14 selected locations where at least 1 mosquito was caught during MRR 5). When comparing the model outcomes to the actual capture data, we note that of the 365 observations (of the number of mosquitoes caught in a given trap at a given time), only 11 observations report numbers outside the 90% credible interval given by the model, providing a good level of confidence on the ability of the model to capture the general dynamic and its ability to make usable predictions both in space and time about the spread and dispersal of WT strain mosquitoes. The reported root-mean-square deviation (RMSE) for this cross-validated experiment is 0.335.

### DSM parameters

The weighted linear pool priors for DSM mortality and diffusion (weighted according to the Bayesian model averaging approach detailed above) are summarised in Table 5. The two parameters are quite different from the WT posterior estimates, with the DSM mortality prior sixfold higher than the WT posterior, and the dispersal multiple orders of magnitude bigger.

**Table 5:** Updated statistics for the PDE model parameters using the WT MRR results and the DSM priors

Parameter	Mean	Q05	Q95
Mortality ($\mu$)^a^	1.05 (0.65)	0.19 (0.17)	2.42 (0.91)
Diffusion (*D*)	753 × 10^3^	8.77	112 × 10^3^

^a^The equivalent daily mortality rate value is given in parentheses, for ease of comparison with other studies

The difference between the posterior distributions for WT and DSM mortality reflects the effect of the Bayesian model averaging but also the significant reduction in uncertainty that occurs when moving from prior to posterior distributions (see Fig. 2), as often occurs in a Bayesian analysis. The linear pool of expert prior distribution for the dispersal distance of WT mosquitoes was also very much higher than its posterior distribution estimated using the data. It is possible therefore that the weighted linear pool prior for DSM dispersal will also prove to be an overestimate, noting of course that the posterior estimates of WT dispersal are conditional on the design of the MRR experiments which was established before this analysis.

### Spread and persistence of male DSM mosquitoes

**Fig. 4:**
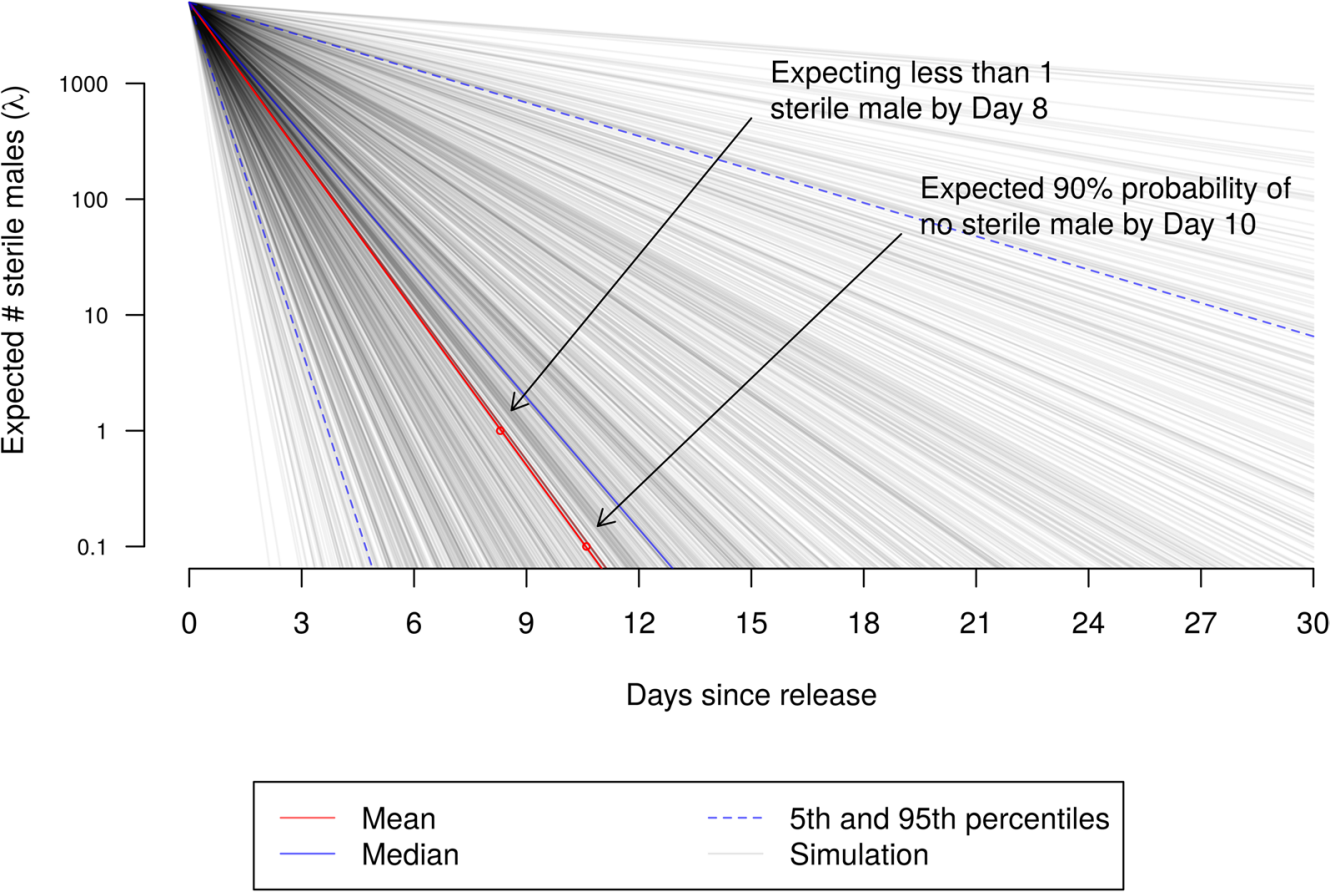
Model prediction of survival. Evolution of the predictive posterior expected number of mosquitoes following a release of 5000. Note that the scale of the *y*-axis is logarithmic, making the model predictions linear

The predicted persistence of DSM mosquitoes following a release of a single cohort of 5000 males is summarised in Fig. 4. The large uncertainty captured in Table 5 is clearly reflected in these predictions: the mean expected survival is estimated to be about 9 to 10 days, with a 90% probability that the expected number of male DSM mosquitoes falls to < 1 by day 10. The 90% credible interval, however, is large, and there is a small probability (c. 0.05) that it could take as long as about 2 months for this to occur.

**Fig. 5:**
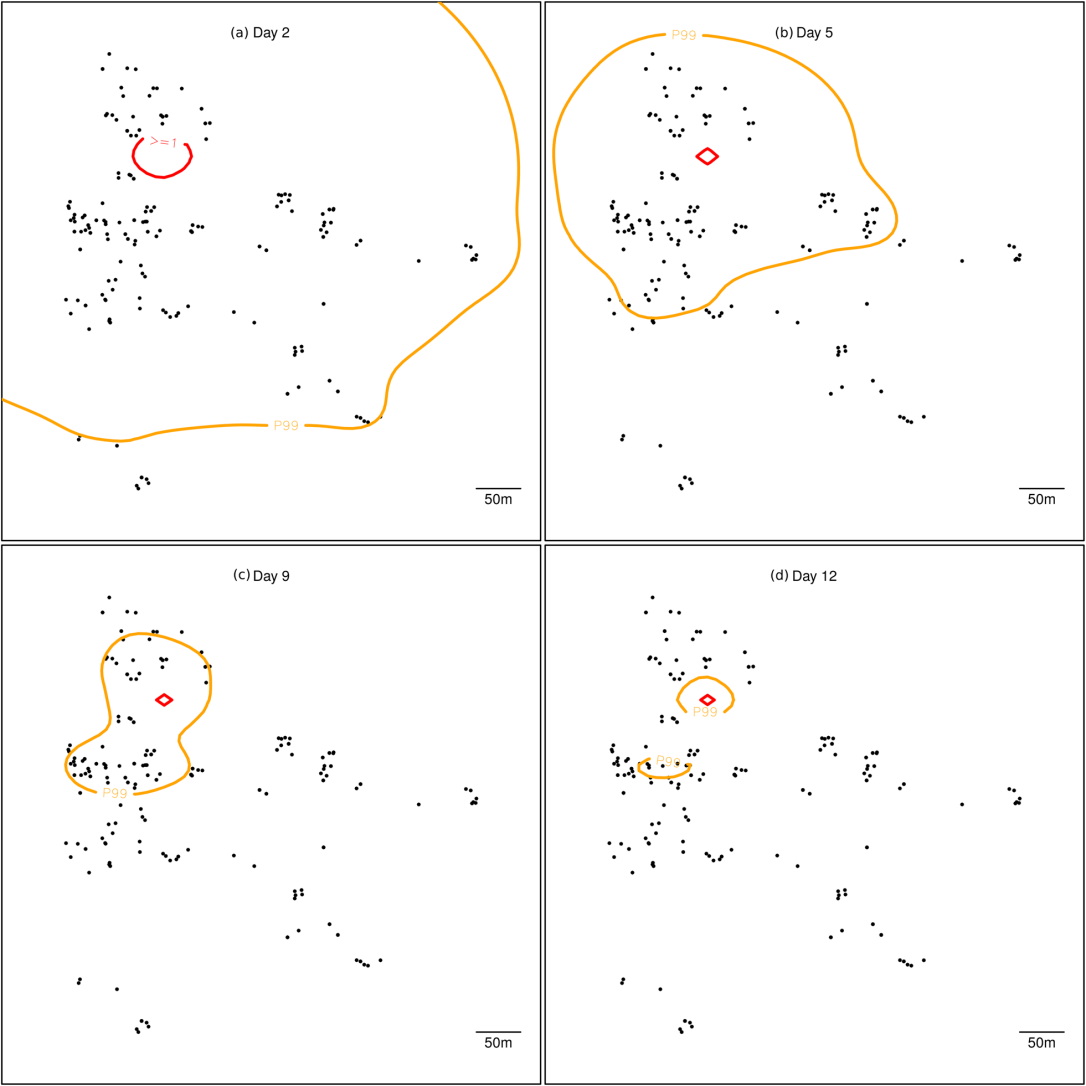
Model prediction of dispersal. Spread of the predictive posterior expected number of mosquitoes following a release of 5000. Orange contour: outside the zone, the probability of finding no DSM mosquito is ≥ 0.99. Red contour: inside the zone, the expected number of DSM mosquitoes is ≥ 1. Black dots indicate compound locations. **a** Extent after 2 days, **b** extent after 5 days, **c** extent after 9 days, **d** extent after 12 days

The dispersal of the DSM mosquitoes is balanced by the mortality rate. Because of this, the DSM are not expected to disperse far from their release site. The expected number of mosquitoes per cell is delineated by the red and orange contours in Fig. 5. For instance, while the spread is expected to extend on average to up to 500 m away from the release location by day 2 (with at most a 1% chance of finding a mosquito further away), the extent then contracts quickly to a limited area (< 250 m from release location) by day 5, to finally about 150 m away from the release location at day 12.

## Discussion

This analysis uses a combination of mathematical modelling, Bayesian inference and expert elicitation to predict the spread and persistence of genetically modified DSM mosquitoes following the release of a single cohort of 5000 individuals. These predictions were made as part of an independent risk assessment [18, 42] (available at: https://targetmalaria.org/resources/library/?category=risks_and-assessments) that also addressed the accidental release of fertile female mosquitoes. The results reported here helped determine the scope of the assessment and in particular provided the rationale for excluding the possibility of effects on non-target organisms caused by changes in food web structures or the provision of ecological services: the predicted survival of the DSM population is too short, and the associated population size too small, to cause any noticeable effect on non-target organisms or ecosystem processes.

The risk assessment was finalised and made public in May 2018, prior to the decision by the National Biosafety Agency (NBA) of Burkina Faso to authorise the field trial (July 2018), but after the submission by Target Malaria of a biosafety application to the NBA seeking authorisation for the release (November 2017; see: https://targetmalaria.org/resources/library/?category=development-pathway). The field release itself occurred in July 2019 (see: https://tinyurl.com/y4qr9lc5). We anticipate that the results from this field experiment will soon be forthcoming and that they will provide an important opportunity to test the accuracy of the predictions presented here. The risk assessment results are case specific and do not generalise to other genetic control strategies that involve (for example) population modification strategies.

In this analysis we were able to validate the model predictions for WT mosquitoes by holding back a proportion of the observation data. This also enabled us to use Bayesian model averaging to identify experts who were more accurate in their prior predictions. By allowing the opinions of these experts to carry greater weight, we were also able to reduce uncertainty in the prior predictions for DSM mosquitoes. This approach assumes, however, that experts who make more accurate predictions about WT mosquitoes will also make better predictions about genetically modified mosquitoes. We will also be able to test this assumption once the results of the DSM field release are published.

Our results demonstrate how field observations greatly reduce the uncertainty between the prior information elicited with independent domain experts and the posterior distribution. Despite their relatively large uncertainty, our experience is that expert-derived prior distributions are essential when attempting to run inference over the multi-dimensional parameter space that dynamic population models demand, and that careful elicitation will therefore continue to be an essential component of future risk assessment studies.

Our posterior estimates of WT dispersal are conditional on a series of MRR experiments that were conducted prior to the elicitation of the dispersal prior distributions. These priors played no part in the experimental design, and the difference between the prior and posterior distributions depends on this design. This difference may reflect conservative prior estimates of mosquitoes’ mean daily dispersal when in the vicinity of attractants (swarm locations), but it may also be an artefact of the MRR design that focussed efforts in and around village compounds to maximise the number of recaptures. As Epopa et al. [20] notes, however, further intensive sampling studies outside or around villages will be useful to ensure that posterior estimates of dispersal are not blind to long-range dispersal outcomes that are not witnessed because of the recapture site design. There is also the possibility of an impact of genetic modification on dispersal, either through life-history traits linked to vagility or perhaps host-seeking behavioural traits. If vagility is negatively impacted, then dispersal could be less for DSM compared to WT mosquitoes, but if host-seeking ability was instead curtailed, then dispersal could perhaps decrease for DSM relative their WT counterparts. As mentioned above, the analysis conducted here could be repeated to take into account the DSM MRR data that were in the planning stages at the time of the risk assessment.

We believe that dynamic population models will form a central component of any risk-based governance system for gene drive-modified mosquitoes. We therefore suspect that confidence in this governance structure will likely depend on the extent to which risk assessments are able to predict the spread and the persistence of mosquitoes carrying gene drive constructs, within the bounds of adequate accuracy. Critically, a staged release strategy provides the opportunity to compare the predictions of these types of models against observed outcomes, learn how to approach the modelling and inference challenges and gradually define the bounds of accuracy that regulators and stakeholders believe are adequate.

This staged learning is borne out by this analysis: the reproductive containment strategy utilised in the DSM mosquitoes provides a good, relatively simple, starting point for this type of analysis. The reaction dynamics in our model are greatly simplified by virtue of the fact that the released male mosquitoes are sterile. The dynamic models for the second stage in Target Malaria’s pathway, namely self-limiting male bias strains, will be more complicated because they must accommodate the birth processes and genetic dynamics that are not relevant here, and also other potentially important interspecific interactions [43]. The models for the third and final stage, that is self-sustaining driving male bias strains, will be more complicated again particularly because the interspecific interactions are likely to be more important and because the spatio-temporal scope of the analysis will be significantly larger.

## Conclusion

Our analysis demonstrates the strength of the Bayesian approach in the context of staged learning. Given its qualities, we believe this paradigm is the most appropriate to handle the prediction and risk assessment challenges that novel, gene drive-based strategies for controlling malaria vectors will pose in the coming years.

## Supplementary Information

**Additional file 1.** Elicitation process, questions and answers.

**Additional file 2.** Derivation of the relationship between the diffusion coefficient and the average dispersal distance.

## Data Availability

The datasets used and/or analysed during the current study are available from the corresponding author on reasonable request. The code used to generate the results is accessible from the following github repository ick003/ rRiskDSMspread.

## Abbreviations

BHM: Bayesian hierarchical model
cfr: Clay pots resting catches
DRAM: Delayed-Rejection Adaptive Metropolis
DSM: Dominant sterile male
GDMMs: Gene-drive modified mosquitoes
MCMC: Markov chain Monte Carlo
MRR: Mark-release-recapture
NBA: National Biosafety Agency
PDE: Partial differential equation
PSC: Pyrethroid spray catches
SS: Swarm sampling
WT: Wild type
